# ArchDB 2014: structural classification of loops in proteins

**DOI:** 10.1093/nar/gkt1189

**Published:** 2013-11-20

**Authors:** Jaume Bonet, Joan Planas-Iglesias, Javier Garcia-Garcia, Manuel A. Marín-López, Narcis Fernandez-Fuentes, Baldo Oliva

**Affiliations:** ^1^Structural Bioinformatics Lab (GRIB-IMIM), Universitat Pompeu Fabra, Barcelona Research Park of Biomedicine (PRBB), Barcelona, Catalonia, 08950, Spain and ^2^Institute of Biological, Environmental and Rural Sciences, Aberystwyth University, SY23 3DA Aberystwyth, Ceredigion, UK

## Abstract

The function of a protein is determined by its three-dimensional structure, which is formed by regular (i.e. β-strands and α-helices) and non-periodic structural units such as loops. Compared to regular structural elements, non-periodic, non-repetitive conformational units enclose a much higher degree of variability—raising difficulties in the identification of regularities, and yet represent an important part of the structure of a protein. Indeed, loops often play a pivotal role in the function of a protein and different aspects of protein folding and dynamics. Therefore, the structural classification of protein loops is an important subject with clear applications in homology modelling, protein structure prediction, protein design (e.g. enzyme design and catalytic loops) and function prediction. ArchDB, the database presented here (freely available at http://sbi.imim.es/archdb), represents such a resource and has been an important asset for the scientific community throughout the years. In this article, we present a completely reworked and updated version of ArchDB. The new version of ArchDB features a novel, fast and user-friendly web-based interface, and a novel graph-based, computationally efficient, clustering algorithm. The current version of ArchDB classifies 149,134 loops in 5739 classes and 9608 subclasses.

## INTRODUCTION

The three-dimensional (3D) structure of a protein is key to determine its function (1,2). In order to exploit this relationship, proteins have been divided and classified according to their fold in databases such as SCOP (3). Structural similarity inferred from these classifications has been used, with different degrees of success, to predict protein functions (4) and interactions (5). Most of these techniques are based on mapping domains over protein sequences via assignation or protein structure modelling (1,3). However, protein domains are also composed of a finite number of secondary structure elements that fit together in a limited number of supersecondary structures (4,6). Supersecondary structures have been used to exploit the structure–function relationship for function and structure prediction (7,8), which has motivated the creation of fragment-based databases such as BriX (9) or SuperLooper (10), protein block identification methods (11,12) and structural alphabets like SA-Mot (13).

Most fragment-based databases split structure fragments according to the number of amino acids involved (i.e. length) and cluster them by means of structural similarity (9). Thus, clusters are limited to fragments of the same length, which allows very little flexibility. On the other hand, methods based on the geometrical relation between two secondary structures have shown a high performance in modelling the aperiodic structure, i.e. loops, connecting them (7,8,14,15).

In a previous work we used the density search (DS) algorithm to combine the geometrical relationship between two secondary structures and the conformation of their linking loop to obtain an automated classification (16). Based on that classification of loops, we have developed ArchDB 2014, which includes super-secondary structures with 3_10_ helices, and a new clustering method that relies on the Markov Clustering (MCL) algorithm (17). This new release of the database still preserves the DS classification in order to maintain consistency with previous database releases. The new database has increased by 5-fold the number of classified loops (from 34 685 to 149 134). Additionally, we have provided a new and intuitive web interface to access the data. We expect this new database to be more useful for the scientific community, in particular for modelling and predicting loop structure and function in proteins. Furthermore, as we have recently showed, the classification of loops can also be employed to predict protein–protein interactions (8,18). Consequently, we expect that this new classification will contribute to improve and extend the prediction of new interactions.

## DATABASE CONTENT

ArchDB classifies loops based on their flanking secondary structures and geometry. The types of secondary structures considered are: β-strands (E), α-helices (H) and 3_10_ helices (G). The geometry of a loop is defined by the distance and the angles hoist, packing and meridian as described in our previous work (14,15). The ontology of a given loop in the classification is therefore defined by its bracing secondary structures (e.g. α-helix–β-strand), its length and its geometry (16).

### Obtaining the loops

Loops were extracted from a non-redundant set of PDB (19) structures with a resolution better than 2.5 Å. Redundancy was removed at 40% sequence identity between PDB chains using CD-HIT (20). The secondary structure of each protein was defined using DSSP (21). Secondary structure was mapped on the corresponding PDB chain sequence when a minimum number of consecutive residues were defined with the same secondary structure type: two, three and four residues for E, G and H, respectively. By this procedure 252 895 different loops were obtained.

### Clustering

The new ArchDB contains two independent classifications based on two different clustering algorithms: DS and MCL. In the previous classification, we used DS to classify loops with similar, but not identical length (using a potential deviation of 1 or 2 amino acids). The large increase of protein structures in the PDB makes the implementation of DS clustering of different-length loops computationally unfeasible. However, a classification of loops that takes into account the flexibility in the definition of the hydrogen-bonding network is very useful for loop modelling. Therefore, we have grouped loops according to their length into four different categories (short, medium, long and extra-long) and we have applied the new clustering algorithm, MCL, to each one of those groups. Furthermore, clustering loops with different lengths allows us to bypass the fact that boundaries of secondary structures are difficult to delineate. For instance, automatic algorithms such as DSSP may fail to accurately define the limits of secondary structures, particularly α-helices (22). The DS clustering has been maintained for consistency with previous releases of the database (16), but this was applied only to classify loops with the same length. See Supplementary Material Methods 1 and 2 for further details on the clustering algorithms.

### Building the classification

**Figure 1.**
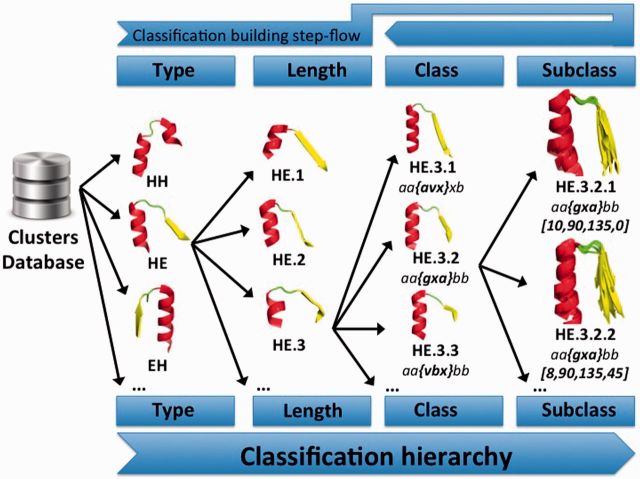
Classification pipeline. Two different methods are applied to build the loop clusters (DS and MCL, see Clustering section and Supplementary Material). Shown within brackets in each subclass is the consensus geometry of the clustered loops, i.e. distance, hoist angle, packing angle and meridian angle [see definitions for loop geometry in the supplementary material, FAQs and in (23)].

A full independent classification is built for each clustering method, i.e. DS and MLC. Each classification is composed of four levels forming a tree-like hierarchy. At the top of the hierarchy, loops are grouped into ‘loop types’, which are defined by its bracing secondary structures (see Obtaining the loops section). Consequently, the first level is composed of 10 loop types: alpha–alpha (HH), alpha–beta (HE), beta–alpha (EH), beta–beta hairpin (BN), beta–beta link (BK), beta–helix3_10_ (EG), helix3_10_–beta (GE), helix3_10_–helix (GH), helix–helix3_10_ (HG) and helix3_10_–helix3_10_ (GG). The second level of hierarchy, in descending order, groups the loops by their length. The MCL clustering approach allows a variation of the loop length (see Clustering section), and thus the length of the cluster is defined by the shortest loop(s). The third level is the class, which is defined by grouping all the clusters with a common conformation of the loop region plus the first two amino acid residues in the bracing secondary structures [defined by the (ϕ,ψ) space and referred as Ramachandran consensus]. The lowest level in the hierarchy is the subclass, which corresponds to the individual clusters (Figure 1). Thus, subclasses within the same class share the same loop conformation but have different geometry. Codes for classes and subclasses are assigned by size (number of loops). This means that the most populated class in a given length will have assigned the code ‘1’ and, similarly, the most populated subclass within a class will be the first one. For example, a subclass labelled as ‘DS.*HH.1.1.1*’ is composed of alpha–alpha (HH) super-secondary structures linked by a loop of one residue, belonging to the most populated class among HH loops of length one and the most populated cluster obtained with the DS approach within this class. The loop classification can be browsed and downloaded through an efficient and user-friendly interface (see Database access section).

### Database statistics

**Figure 2.**
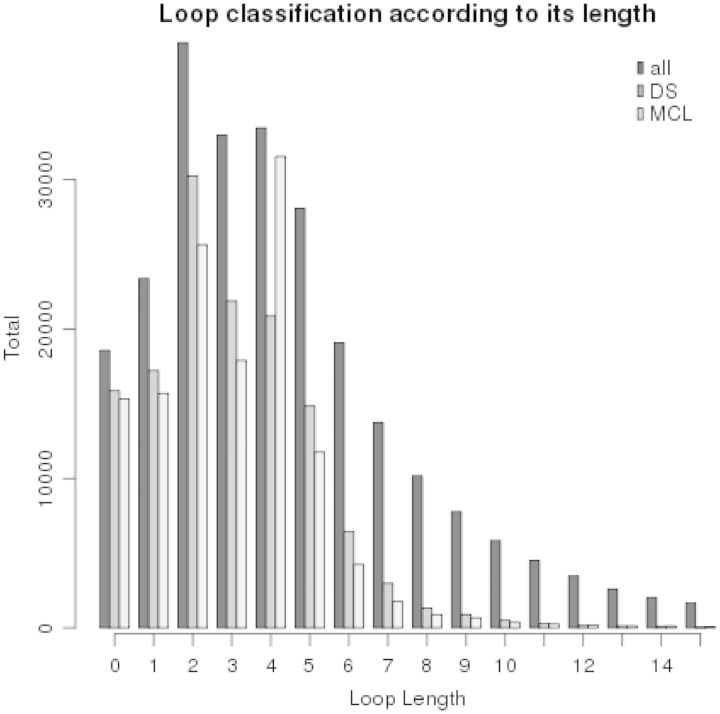
Distribution of classified loops for each of the clustering method as a function of loop length.

A total of 252 895 loops were extracted from a set of 13 238 non-redundant proteins (see Obtaining the loops section). Loops are unevenly distributed among the different types, and only ∼50% of them could be classified with each method. The highest percentage of loops classified had short or medium lengths. Two different reasons can be identified as probable causes for this behaviour: (i) the larger number of loops accumulated at shorter lengths and (ii) the smaller number of degrees of freedom in the conformational space of short or medium length loops (Table 1, Figure 2). This observation also agrees with our previous work showing the saturation of loop conformations for short and medium loops (24).

**Table 1.** The different loop types according to their flanking secondary structure

Type	Type description	All	DS (%)	MCL (%)
BK	β-link	28 418	11 777 (41.4)	6054 (21.3)
BN	β-hairpin	35 616	27 995 (78.6)	22 536 (63.3)
EG	β-helix3_10_	18 349	6950 (37.8)	8531 (46.5)
EH	beta–alpha helix	42 442	23 364 (55.0)	19 661 (46.3)
GE	helix3_10_–beta	16 478	6829 (41.4)	7731 (46.9)
GG	helix3_10_–helix3_10_	3498	704 (20.1)	23 (0.6)
GH	helix3_10_–α-helix	16 249	7537 (46.9)	10 141 (62.4)
HE	α-helix–β	42 079	24 870 (59.1)	23 327 (55.4)
HG	α-helix–helix3_10_	14 472	5689 (39.3)	9133 (63.1)
HH	α-helix–α-helix	35 294	18 200 (51.5)	19 503 (55.2)

The total number for each type as well as the number of each type that has been classified is also shown.

**Figure 3.**
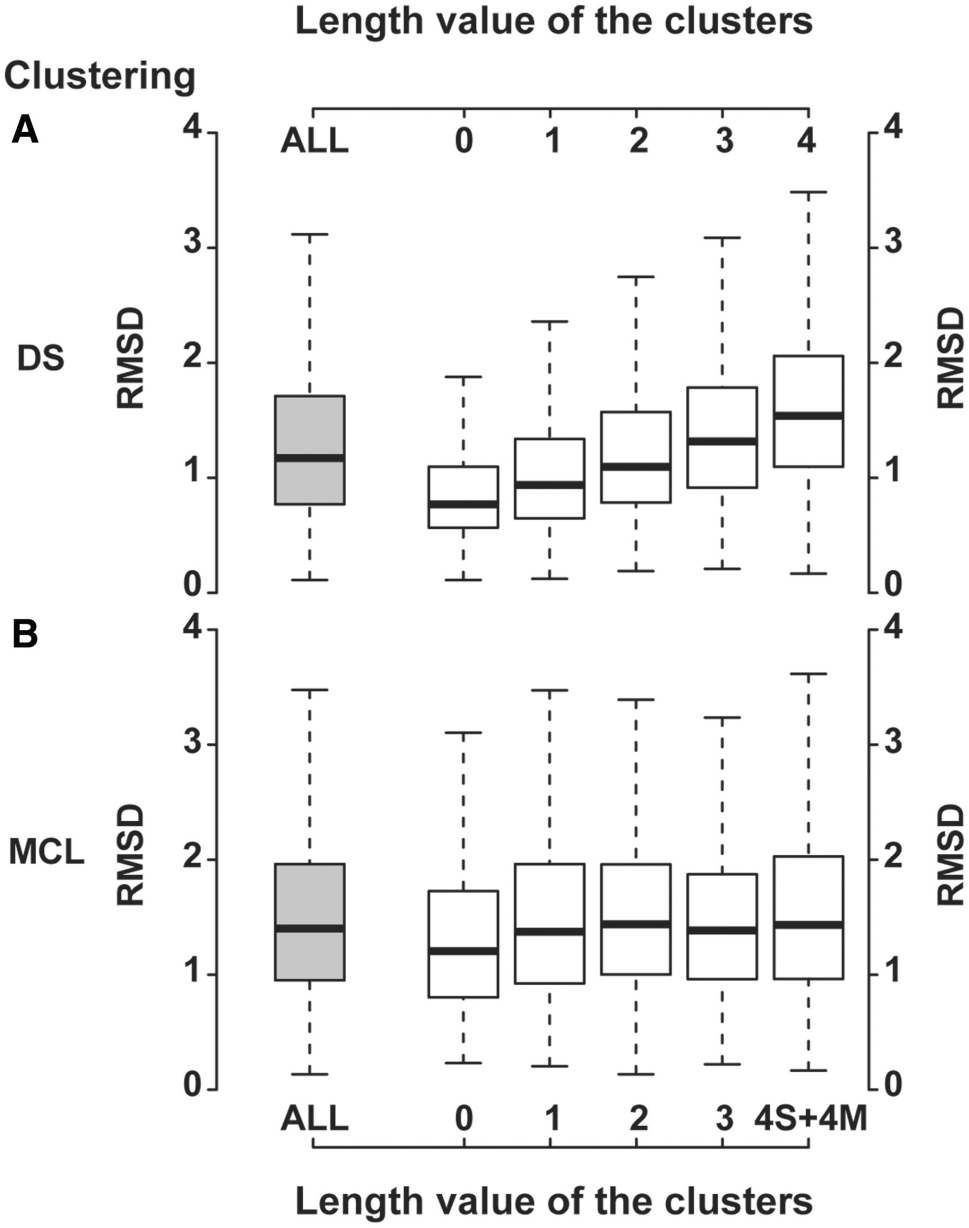
RMSD distribution of the five most populated loop lengths (from 0 to 4) for all loop types. Distribution using DS clustering (top). Distribution using MCL clustering (bottom; this includes two types of subclasses 4S and 4M at length 4). See Supplementary Figures S1 and S2 for a detailed analysis of the RMSD distribution by type-length.

The clustering of loops is RMSD-independent and, thus, this measure can be used *a posteriori* as an indication of the quality of the clustering. The RMSD values of the loops of each cluster were obtained with a structural alignment using STAMP (25). The distribution of RMSD as a function of the loop length is shown in Figure 3 (see Supplementary Figures S1 and S2 for details on each type of loop). The MCL algorithm clusters loops of different lengths, resulting in slightly higher RMSD measures than the ones obtained using the DS algorithm. Still, the average RMSD is below 1.5 Angstroms. Even with different loop lengths, the distribution of RMSDs when using the MCL algorithm is similar to the distribution obtained with DS algorithm using fixed loop lengths (Figure 3, Supplementary Figures S1 and S2).

### Applications of the database

The previous ArchDB classification of loops was used as gold standard to develop new methods for loop prediction [e.g. (26)], as a test set in support-vector-machine methods for the identification of β-hairpins (27), to search templates for protein modelling (15), for function prediction (28), evolutionary conservation (29) and, more recently, to understand and predict protein–protein interactions (8,18). The new database provides new insights useful for researchers focused on the structural/functional features of protein loops [see Example 1 on the P-loop in Supplementary Material; (30)] and improves the prediction of the structural conformation of loops (by increasing the coverage of loop conformations and the possibility to search among different loop-lengths). Moreover, the annotation of external databases to the classes and subclasses of loops, such as SCOP (3), GO (31), ENZYME (32) or DrugBank (33), and the analysis of interacting heteroatoms and known PDB sites, will help researchers on the annotation of protein function. Finally, the extension of the database of loops will also help to improve the coverage on predictions of protein–protein interactions, the detection of enabling/disabling loops (7) and the annotation of binding sites.

## DATABASE ACCESS

The database is available in the form of a user-friendly web interface at http://sbi.imim.es/archdb. The classification is accessible through a composed panel, which allows users to visualize the entire hierarchy, i.e. loop type, loop length, class and subclass, while the selected data is shown in the main section of the web page. There are different visualization modes for every step of the classification. Clustering, type and length views offer useful statistics of the loops included at each level, while class and subclass views offer detailed information that defines such levels. The alignment of the sequence, the secondary structure calculated with DSSP, and the (ϕψ) angles defining the conformation of each loop [in codes as in (16)] is provided in the details of the subclass. External annotations of databases, functional sites from PDB and heteroatoms found at distance shorter than 6 Å from the atoms of the loops, are also shown in the detailed information of the subclass. The enrichment of functions [in GO terms (31) and ENZYME EC codes (32)], drug targets [defined by DrugBank (33)] and SCOP domains (3) provides a useful mechanism to annotate the subclass and infer a putative relationship between function and local structure. Additionally, a downloadable section provides the user with a tab-formatted file containing the most relevant data of the classification for local use. Finally, a Frequent Asked Questions section provides guidance on browsing and understanding the database. In some relevant views (loop and subclass), the web provides 3D visualizations both for each individual loop and for the structural superposition [build with STAMP (25)] and visualization of loops within the subclass.

## SUPPLEMENTARY DATA

Supplementary Data are available at NAR Online, including [34].

## Funding

Spanish Ministry of Science and Innovation (MICINN) [FEDER BIO2008-0205, FEDER BIO2011-22568, EUI2009-04018]; FI-DGR 2012 fellowship from ‘Generalitat de Catalunya’ (to M.A.M.L.). Funding for open access charge: Spanish Ministry of Science and Innovation.

*Conflict of interest statement*. None declared.
